# Special Issue “Molecular Genetics, Breeding, and Multi-Omics Approaches in Rice Research”

**DOI:** 10.3390/ijms272110034

**Published:** 2026-11-09

**Authors:** Longxing Tao, Tingting Chen

**Affiliations:** State Key Laboratory of Rice Biology and Breeding, China National Rice Research Institute, 359 Tiyuchang Road, Hangzhou 310006, China; taolongxing@caas.cn

Rice (*Oryza sativa* L.) is the primary staple crop feeding over half of the global human population, and integrated research on rice breeding and cultivation constitutes an indispensable cornerstone for safeguarding worldwide food security. In the context of ongoing global changes, agricultural production is confronted with multiple severe constraints, including continuously shrinking arable land resources, steady population expansion, and the rising frequency of destructive extreme climatic events such as high-temperature episodes, seasonal drought and soil salinization. Under such pressures, developing elite rice cultivars characterized by high grain yield, desirable eating and appearance quality, as well as broad-spectrum abiotic and biotic stress resilience, has become an urgent priority for modern rice agriculture. Breeding innovation exploits natural genetic diversity through germplasm excavation, genetic dissection and artificial variety creation. Milestone achievements including semi-dwarf green-revolution breeding and successive generations of three-line and two-line hybrid rice have dramatically increased rice yield potential, fully demonstrating the enormous production value of genetic improvement. Even the most favorable genetic backgrounds, however, cannot reach their full agronomic potential without scientifically matched field cultivation practices. Rational agronomic management optimizes source–sink–flow carbon distribution, fine-tunes water and fertilizer input, and implements targeted stress mitigation, effectively converting intrinsic genetic advantages into stable on-farm grain output. Furthermore, advanced green low-carbon cultivation strategies can reduce chemical fertilizer and pesticide inputs and alleviate greenhouse-gas emissions from paddy ecosystems, reconciling high-yield production goals with ecological sustainability.

Recent decades have brought remarkable methodological advances that profoundly reshape both rice breeding and cultivation research systems. Within breeding research, conventional sexual cross-breeding still functions as the foundational framework for assembling comprehensive elite genetic backgrounds. Meanwhile, molecular-assisted breeding tools have greatly improved selection accuracy and research efficiency. Marker-assisted selection and favorable-allele pyramiding permit reliable genotype-based screening in early plant generations, effectively eliminating confounding noise caused by environmental fluctuations. Genotypic prediction models constructed from multiple trait-associated alleles, e.g., linear regression models for plant-height forecasting, provide practical decision-making support for breeders. The CRISPR-Cas9 genome-editing toolkit enables targeted gene knockout and base editing to generate novel allelic variations, drastically shortening the cycle for creating improved germplasm. Rapidly expanding multi-omics resources, including pan-genome datasets, genome-wide association studies (GWAS), transcriptomics, proteomics and metabolomics, support large-scale identification of QTLs and candidate genes underlying yield, grain quality and stress responses, allowing systematic dissection of complex developmental and stress-regulatory networks. Genomic-selection approaches further push breeding practice from phenotype-driven screening toward genotype-oriented precision improvement. In the field of cultivation research, diverse green and labor-saving cultivation technologies have been progressively optimized including water-saving dry direct-sowing systems, biodegradable-film coupled precision irrigation, and cold-region film-mulching transplanting techniques, which well balance grain productivity with resource conservation. Stress-physiology-driven agronomic strategies have been established to mitigate yield and quality losses triggered by heat, drought and salinity, by modulating reactive-oxygen-species homeostasis and cellular energy metabolism. Integrated ecological pest and disease management combining biopesticides and optimized aerial spraying protocols adapts to mechanized large-scale rice production. Notably, increasing studies combine cultivation physiology with multi-omics profiling to uncover molecular regulatory changes triggered by different agronomic treatments, driving cultivation research beyond simple phenotypic observation toward multi-dimensional systematic interpretation of trait-formation mechanisms.

Notwithstanding these encouraging technical developments, a series of prominent bottlenecks continue to hinder the translation of basic knowledge into real-world rice production benefits. Most economically vital traits such as grain yield, comprehensive grain quality and multi-stress tolerance belong to polygenic quantitative traits governed by numerous minor-effect loci, whose phenotypic performance is heavily modulated by complicated genotype-by-environment interactions. A large number of candidate genes uncovered by omics and laboratory molecular experiments exhibit impressive effects under controlled conditions yet fail to deliver expected agronomic gains under variable multi-location field environments, creating a noticeable disconnect between fundamental molecular research and practical breeding applications. Although considerable numbers of new rice varieties are officially released every year, truly breakthrough cultivars that simultaneously realize high yield, superior grain quality and durable multi-stress resistance remain relatively scarce, while valuable allelic resources harbored in wild rice accessions and local landraces are far from being sufficiently mined and utilized. For cultivation research, many newly bred cultivars exhibit limited genetic variation as a result of tailored agronomic guidelines, so their genetic advantages cannot be fully expressed under local farmer-field conditions. A substantial proportion of stress-physiology experiments are carried out within greenhouse-controlled settings, whereas field investigations targeting naturally occurring combined multiple stresses are still limited. Integrated research frameworks that holistically consider genotype–agronomic–practice–environment interactions are still insufficient.

Against this backdrop, we launch the Special Issue entitled “Molecular Genetics, Breeding, and Multi-Omics Approaches in Rice Research”. This Special Issue aims to gather cutting-edge original research papers and authoritative reviews that bridge molecular genetic dissection, breeding innovation and multi-omics analytical strategies. It intends to assemble scattered research outputs under one thematic framework, clarify the molecular regulatory mechanisms governing yield, grain quality and stress-adaptation traits, summarize feasible technical pipelines for translating omics-derived candidate information into breeding practice, and highlight existing bottlenecks restricting the transformation of basic research into field production. By bringing together findings from diverse research teams worldwide, this Special Issue seeks to strengthen academic exchange between molecular geneticists, breeders and cultivation researchers, inspire integrated thinking of genotype–environment–agronomic management, and provide theoretical references and technical inspirations for developing high-yield, high-quality and climate-resilient rice germplasm, ultimately contributing to global food-security advancement.

Lee et al. [contribution 1] investigated cell-wall invertase-encoding genes *OsCIN1* and *OsCIN2*, which are specifically expressed in anther maternal tissues rather than in pollen grains. Genetic analyses using *oscin1*/*oscin2* double mutants revealed sporophytic male sterility with only approximately 2% seed-setting rate as well as shrunken, opaque seeds, whereas single mutants displayed no obvious reproductive defects, indicating functional redundancy between *OsCIN1* and *OsCIN2*. Genetic crossing assays demonstrated that both pollen-development defects and shrunken-seed phenotypes were determined by maternal-tissue genotypes instead of filial or pollen genotypes. Enzyme-activity measurements showed that CIN activities in wild-type anthers and seeds were over 500-fold and 5-fold higher than those in leaves, respectively. *Oscin1*/*2* double mutants exhibited greatly reduced CIN activity in reproductive organs. Consistent with enzyme changes, the double mutant had decreased hexose and starch contents in anther locular fluid and developing seeds. The complementation test with 35S-driven *OsCIN1* could largely restore seed fertility yet failed to fully rescue seed opacity. Thus, the study indicated that maternal-tissue-originated *OsCIN1* and *OsCIN2* modulate apoplastic sucrose flux, maintain carbohydrate supply for pollen and seed development, and their activity is fine-tuned by tissue-specific expression and potential post-translational regulation to shape carbon partitioning in rice reproductive tissues.

Su et al. [contribution 2] investigated yield-formation physiological mechanisms using two early-rice cultivars, Zhongzu18 (high-yield) and Yongxian15 (control). Zhongzu18 exhibited significantly higher seed-setting rate, 1000-grain weight, theoretical and actual grain yield, together with better grain quality featuring higher head-rice rate and lower chalkiness degree and chalky-grain rate. At maturity, Zhongzu18 accumulated more total dry matter and allocated a larger proportion of dry mass to panicles, indicating more efficient translocation of assimilates towards grains. Although SPAD values were comparable between varieties, Zhongzu18 maintained significantly higher flag-leaf net photosynthetic rate and actual fluorescence quantum efficiency after anthesis. Higher starch concentration was detected in panicles of Zhongzu18 at late grain-filling stage. The sucrose-transport-related gene *SUT1* showed markedly elevated expression across leaf, stem-sheath and panicle tissues in Zhongzu18. Moreover, Zhongzu18 possessed higher ATP and ATPase levels in multiple organs, enhanced antioxidant-enzyme activities and lower malondialdehyde (MDA) content than Yongxian15. Thus, the study indicated that strengthened photosynthetic capacity, improved cellular energy status and superior antioxidant capacity jointly up-regulate sucrose-transport gene expression, facilitate assimilate translocation to sink organs, delay leaf senescence, and consequently achieve higher grain yield and better grain quality in short-growth-duration early rice.

Huang et al. [contribution 3] developed linear-regression prediction models for rice plant height based on pyramiding height-related alleles, using an association-mapping panel of 273 rice varieties and validated with a 219-line recombinant inbred line (RIL) population. Twenty-two molecular markers significantly associated with plant height were identified via mixed-linear-model association analysis across multiple environments. Both the association panel and RIL population showed that plant height increased with the number of height-increasing alleles pyramided, confirming the additive effect of these loci. Linear regression models built with 2–10 marker loci revealed that prediction accuracy increased with the number of loci (up to five loci). Models built with 5–10 loci yielded average absolute prediction errors ranging from 11.05 to 11.96 cm, which were comparable to natural environmental-caused height variation (5.72–12.79 cm). Two-way ANOVA revealed that genotype contributed 76.44% of height variance, genotype-by-environment interaction accounted for 19.95%, while environment alone contributed only 3.61%. Thus, the study indicated that the multi-allele-based linear regression model can reliably predict rice plant height and provides a practical tool for marker-assisted rice breeding, which may inspire similar prediction strategies for other crop quantitative traits.

Tran et al. [contribution 4] characterized the biochemical properties and in vivo physiological functions of rice class-I peroxygenase-9 (OsPXG9). In vitro kinetic and LC-MS/MS analyses demonstrated that OsPXG9 preferentially metabolizes substrates from the lipoxygenase-9 (9-LOX) oxylipin pathway rather than the 13-LOX pathway. It utilizes hydroxy fatty acids as oxygen acceptors instead of free unsaturated fatty acids, and shows higher catalytic efficiency with cumene hydroperoxide than hydrogen peroxide. OsPXG9 catalyzes intra- and intermolecular epoxidation reactions to produce epoxy- and trihydroxy-fatty-acid derivatives. Two *OsPXG9*-overexpressing transgenic rice lines (ox2-1, ox2-2) exhibited enhanced tolerance to drought and salt stress. Under abiotic stress conditions, overexpression lines accumulated less reactive oxygen species (ROS), lower MDA levels and fewer apoptotic cell deaths compared with wild-type plants. Under non-stressed conditions, the highly overexpressed ox2-1 line displayed repressed transcript levels of antioxidant-related genes (*OsAPX1*, *OsCAT-A*, *OsSOD1*, *OsGR*). Upon drought-salt treatment, these antioxidant genes were induced. CRISPR-Cas9-mediated knockout attempts of *OsPXG9* failed to obtain viable mutants, implying its essential role for callus regeneration. Thus, the study indicated that OsPXG9 scavenges lipid hydroperoxides and modulates cellular ROS homeostasis, negatively regulates ROS accumulation, and improves rice cellular resistance against drought and salt stress via crosstalk with other antioxidant pathways.

Nguyen et al. [contribution 5] investigated the biological effects of constitutive overexpression of *OsCIN1*, which encodes a rice apoplastic cell-wall invertase. Subcellular localization confirmed OsCIN1 resides in the apoplast. Promoter-GUS assays showed *OsCIN1* is preferentially expressed in vascular tissues, especially ovular vascular and lateral stylar vascular traces during seed development. Transgenic 35S-driven *OsCIN1* overexpression (OX) lines exhibited substantially elevated CIN enzymatic activity in both vegetative and reproductive tissues. Flag-leaf sugar profiling at flowering revealed nearly complete sucrose depletion, accompanied by significant accumulation of hexoses and starch in OX plants. Under paddy-field conditions, OX lines displayed impaired agronomic traits: tiller number was reduced by about 50%, together with decreased 1000-grain weight, grain width and thickness. Constitutive high CIN activity throughout the plant disrupted the apoplastic sucrose concentration gradient required for long-distance phloem transport; hexoses generated by excessive hydrolysis cannot be transported over long distances and accumulated in source tissues, resulting in insufficient carbon supply for sink organs. Thus, the study indicated that simple constitutive overexpression of *OsCIN1* cannot improve grain yield; precise spatiotemporal control of cell-wall invertase activity is essential to remodel source–sink carbon partitioning and boost rice productivity.

The five studies included in this Special Issue uncover source–sink carbon partitioning, abiotic-stress adaptation, quantitative-trait prediction and molecular regulatory mechanisms in rice, covering cell-wall invertase function, peroxygenase-9-mediated stress resistance, multi-omics-related high-yield physiology, QTL-based plant-height prediction, and spatiotemporal control of carbon-metabolism-related genes. Functional gene studies include two reports focusing on apoplastic sucrose flux and oxidative stress response. Nguyen et al. [contribution 5] demonstrated that constitutive overexpression of apoplastic-localized *OsCIN1* disrupted apoplastic sucrose gradients, depleted leaf sucrose, elevated hexose and starch accumulation, and caused reduced tiller number and decreased grain weight, highlighting the necessity of precise spatiotemporal control for cell-wall invertase activity. Tran et al. [contribution 4] characterized rice class-I OsPXG9, which preferentially metabolizes 9-LOX-pathway oxylipin substrates. *OsPXG9* overexpression scavenged reactive oxygen species, lowered MDA accumulation and apoptotic cell death, and enhanced drought and salt tolerance via crosstalk with other antioxidant pathways. Two further works addressed yield-related physiology and quantitative-trait prediction. Su et al. [contribution 2] compared two early-rice varieties and revealed that strengthened photosynthetic capacity, improved cellular ATP-ATPase energy status, activated antioxidant enzymes and up-regulated sucrose-transport-related *SUT* genes collectively promoted assimilate translocation, bringing higher seed-setting rate, 1000-grain weight and better grain quality. Huang et al. [contribution 3] performed association-mapping using 273 rice accessions, identified 22 plant-height-associated markers, and constructed linear-regression prediction models based on pyramided height-increasing alleles. Validation in an RIL population showed prediction errors of 11.05–11.96 cm for 5–10-locus models, comparable to naturally caused environmental variation in height (5.72–12.79 cm), confirming the feasibility of genotype-based plant-height prediction for marker-assisted breeding. In addition, Lee et al. [contribution 1] characterized maternal-tissue-expressed *OsCIN1* and *OsCIN2*. The *oscin1 oscin2* double mutant exhibited severe male sterility and shrunken opaque seeds, maternal-origin *OsCIN1*/*OsCIN2* maintained apoplastic sucrose flux for anther and seed development, modulating carbohydrate supply for reproductive-organ formation. In summary, these studies advance our understanding of rice source–sink dynamics, oxidative-stress homeostasis and quantitative-trait genetic architecture. From multiple dimensions including enzyme biochemistry, physiological phenotyping, multi-allele pyramiding and genetic-material validation, they reveal how maternal-tissue-specific carbon-metabolism genes, antioxidant systems and additive QTL effects jointly shape rice reproductive performance, stress resilience and agronomic traits, supplying theoretical support and molecular resources for modern rice molecular breeding.

